# E-scooter driving under the acute influence of alcohol—a real-driving fitness study

**DOI:** 10.1007/s00414-022-02792-3

**Published:** 2022-02-26

**Authors:** Katharina Zube, Thomas Daldrup, Michael Lau, Rüdiger Maatz, Anne Tank, Irina Steiner, Holger Schwender, Benno Hartung

**Affiliations:** 1 Institute of Legal Medicine, University Hospital Düsseldorf, Düsseldorf, Germany; 2 Institute of Mathematics, Heinrich Heine University, Düsseldorf, Germany; 3 Federal Court of Justice, Karlsruhe, Germany; 4 Institute of Legal Medicine, Ludwig-Maximilians-University, Munich, Germany

**Keywords:** E-scooter, Driving under the influence, Neurological examination, Alcohol, Impairment

## Abstract

**Purpose:**

To assess the effects of alcohol on the ability to drive an e-scooter, driving tests reflecting real-life situations accompanied by medical examinations focusing on balance were conducted at different blood alcohol concentrations (BACs).

**Methods:**

Fifty-seven subjects who consumed alcohol (28 female, 29 male) and 6 consistently sober subjects (3 female, 3 male) participated in the study. Alcohol was administered on a fixed schedule, and the individual drinking quantity was individually calculated in advance using the Widmark formula. Repeated runs through a fixed course were performed. Following each ride, a blood sample was taken for BAC determination, and medical tests were performed.

**Results:**

Even at low BACs (0.21–0.60 g/kg), subjects showed a significant decrease in driving performance, to approximately 60% of the initial level. Differences in driving performance at different BAC ranges were observed for different obstacles, especially for the narrowing track, gate passage, slalom, and driving in circles obstacles. Furthermore, worse Romberg and Unterberger test results were correlated with worse driving performance. It cannot be assumed that learning effects during the study had a relevant effect, as shown in the comparison of the driving performance of the alcohol-consuming group with that of the control group. Sex-specific differences were not found.

**Discussion:**

Significant deteriorations in driving performance at BACs below 1.10 g/kg confirmed alcohol-related risk potential when using e-scooters. At this time, these findings may lead to the assumption of “relative driving impairment” in Germany. The Romberg and Unterberger tests could be considered a complementary investigation method for the assessment of e-scooter driving impairment.

**Conclusion:**

Even at rather low BACs between 0.21 and 0.40 g/kg, there was a significant deterioration in driving performance under the influence of alcohol compared to sober, which highlights the negative effects of alcohol on e-scooter driving.

**Supplementary Information:**

The online version contains supplementary material available at 10.1007/s00414-022-02792-3.

## Introduction

Due to the increasing interest in e-scooters, an increasing number of legislative problems have become apparent [1]. I.a., the question arose, whether e-scooter driving under the influence of alcohol is comparable to driving of other motor vehicles or if it constitutes a new category. The driving of e-scooters differs from the driving of other motor vehicles, as it takes place in an upright standing position, as e-scooters have low wheel diameters and short steering bars and usually possess the ability to accelerate quickly [2, 3]. One could imagine that the sense of balance must meet higher demands for the driving of e-scooters than for the driving of other vehicles. After alcohol consumption, these are particularly important factors that must be evaluated specifically.

The negative impact of alcohol on driving a car or bicycle is well known [4, 5]. A distinction must be made between constraints in simple and complex actions [5, 6]. Moreover, it is necessary to assess interindividual differences in driving performance [6].

Furthermore, e-scooters are mostly used as independent alternatives to cars and bicycles, often especially for the last kilometres to a destination [7]. “Fun” is another main reason for driving e-scooters [8, 9]. According to the Federal Statistical Office (“Statistisches Bundesamt”), many drivers in e-scooter accidents in Germany in 2020 were under the influence of alcohol (18.3%) [10, 11]. Twenty-nine percent of these accident-involved drivers were in the age range of 15–25 years [11]. Few studies have investigated and noted the time of accidents. Accidents often happen during the weekend and at night [3], often under the influence of alcohol [12].

Injuries related to e-scooter accidents mostly affect the head and face and the upper extremities [3, 7, 12–16]. Uluk et al. [17] stated that these injury patterns are similar to those of cyclists and pedestrians, but the accident mechanisms are different. Some of the patients in this study claimed to have lost balance while making a hand signal for turning [17]. Störmann et al. [14] compared the injury patterns to those for different types of sports and determined that the injury patterns were similar to those of skateboarders and snowboarders [14, 18, 19]. In a known manner, these sports have a high demand for a sense of balance. In addition, electric motor acceleration could increase the risk of accidents [20]. The consumption of alcohol could affect these particular factors which would increase the safety risk.

The alcohol limits for the operation of an e-scooter vary. In some countries, the limits are the same as those for riding a bicycle; in others, the limits are the same as those for riding e-bikes; and in other countries, the limits are the same as those for driving motor vehicles [21].

In Germany, in addition to the offence limit of a blood alcohol concentration (BAC) of 0.50 g/kg, a sophisticated system of alcohol thresholds exists. The jurisdiction differentiates between “relative” and “absolute” impairment for both motor and nonmotor vehicles. A “relative” impairment citation may be given starting at 0.30 g/kg if alcohol-related driving errors or relevant alcohol-related psychophysical impairments are evident. An “absolute” impairment citation may be given if a driver has a BAC of at least 1.10 g/kg at the time of an incident. Practical driving resp. cycling tests are crucial for the determination of BAC thresholds [4].

The main objectives of this study were to examine alcohol-related impairments when using an e-scooter, with the question of whether the current BAC limit of 1.10 g/kg for absolute impairment to drive an e-scooter is adequate.

The control group should disclose whether repeated driving over several hours leads to an obstacle habituation with an improvement in the driving performance or exhaustion with worsening of the driving performance.

## Material and methods

The study was approved by the ethics committee of the University Hospital Düsseldorf (study number: 2019–735). All involved persons were contractually insured.

### Inclusion criteria

The following inclusion criteria had to be met for participation in the study:

o age between 18 and 50 years,
p ability to drive an e-scooter (at least 2 e-scooter utilisations before the study),
q experience with the consumption of alcohol (the consumption of at least two alcohol drinks per month in the past 6 months),
r negative urine screening for drugs prior to the start of trial, and
s medical health certificate (at most 4 weeks old).

#### Exclusion criteria

o pregnancy or breastfeeding;
p general alcohol abstinence;
q neurological or psychiatric diseases;
r disturbances of liver function or of the muscular or skeletal system; and
s a history of joint-preserving or joint-replacing procedures.

### Course

The course was partially based on the templates introduced by Schewe et al. [22, 23] and Hartung et al. [4], as well as new elements that were considered potentially relevant for e-scooter drivers. Previously described obstacles included a narrowing track, a gate passage, an alley drive, driving in circles counterclockwise, and a slalom ride with decreasing spacing.

**Fig. 1:**
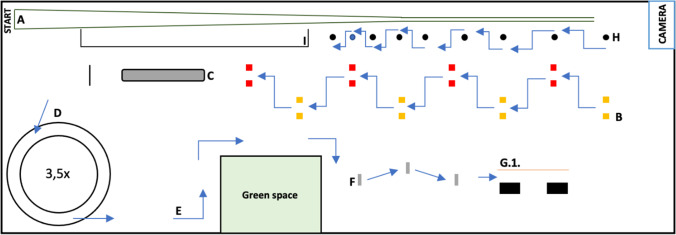
Course; A: narrowing track (45 m length); B: gate passage (spaced at 1.30 m); C: gravel bed (6.90 m length); D: driving in circles counterclockwise 3.5 times; E: three turns with timely directional indication (left–right-right); F: three thresholds; G.1: alley (width: 1.05 m; length: 5.55 m); H: slalom ride with decreasing spacing (2 × 4 m; 2 × 3 m, 2 × 2 m; 1 × 1.5 m); I: speed track (17.7 m resp. 16.5 m)

On the first and second day, a fixed lap with 9 obstacles had to be passed (Figure 1). The following sequence had to be completed: A, B, C, D, E, F, G.1, H, I.

**Fig. 2:**
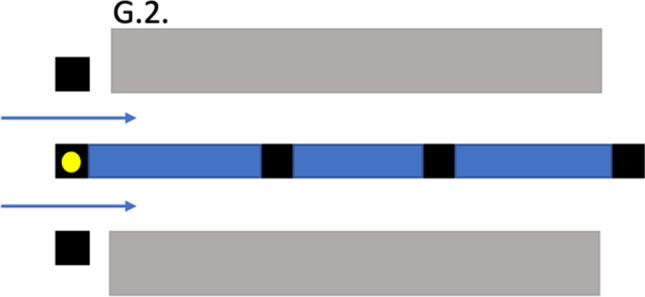
G.2; alley drive with an upstream light signal indicating which of the two lanes should be used

Two obstacles were varied on days 3 and 4: the single alley drive (G.1) became an either/or-alley drive. In this section, the drivers had to react to a previously emitted light signal (Figure 2; G.2.). Furthermore, the speed track (I) was shortened from 17.7 to 16.5 m.

Moreover, on days 3 and 4, 6 elements had to be passed a second time after the completion of the abovementioned course (Figure 1; elements with two passes in the following order A, B, C, D, E, F, G.2, H, I, D, E, F, G.2., B, C). Passing the elements A–I took approximately 1:40 min.

### E-scooters and test area

E-scooters (Tier, model ES 400B, Tier Mobility AG, Berlin, Germany) were rented. The maximum speed that could be achieved was 20 km/h (German legislation).

The study took place at a closed, nonpublic, asphalted resp. paved area.

There were a total of four test days with dry, wet, and rainy weather conditions.

The prescribed protective clothing was a jet helmet, motorcycle clothing with integrated elbow and spine protectors, and additional hip, knee, and wrist protectors.

### Cameras

Driving was recorded with two video cameras from different perspectives: a film camera in an elevated position for overview recording and a mobile camera (GoPro Hero 3) mounted below the handlebars and facing the front wheel.

### Alcohol

Each alcohol-consuming subject had to choose one of the following alcohol types in advance: beer, red wine, white wine, vodka, grain spirits, or rum. Switching during the experiment was not permitted.

### Basic experimental set-up

Each test subject was allowed to run the course until he/she felt confident and familiar with it (at least three times).

This was followed by the first run (sober), which defined the baseline.

The following alcohol consumption period varied between 02:22 and 04:41 h.

All subjects completed at least three runs in the state of alcoholisation, one of which was approximately at the assumed maximum BAC.

**Fig. 3:**
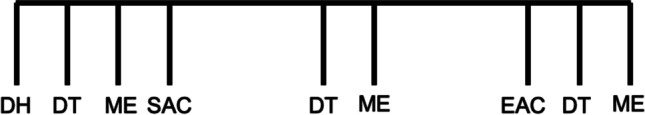
Timeline of basic experimental set-up. DH, driving habituation; DT, driving test; ME, medical examination (including blood sample); SAC, start of alcohol consumption; EAC, end of alcohol consumption. The alcohol consumption time varied between 02:22 and 04:41 h. Between ACB and ACE were one up to two DT completed. After ACE were two up to three DT completed. The time from DH to the last ME varied between 05:15 and 07:30 h (excluding drop outs)

After each run (both in the state of soberness and under the influence of alcohol), a blood sample was taken, and medical examinations of the sense of balance were performed (Romberg test, Unterberger test, see below). The timeline is presented in Figure 3.

Before each run, test subjects were named a trisyllabic word, which had to be reproduced at the end of the course.

The individual drinking quantity was calculated in advance for each subject by using the Widmark formula. A resorption deficit was taken into account, which was set at 20% for beer and 15% for wine, vodka, grain spirits, and run. The proportion of body water was adjusted to the respective body weight, height, and sex. The calculations were made in such a way that the highest BAC at which a driving test should be performed at approximately 1.30 g/kg.

### Medical examinations

After each ride, blood samples were drawn, and both the Romberg test and Unterberger test were carried out. The examinations took place in a slightly darkened room that was as quiet as possible so that the possibility of orientation to light or hearing sources was minimised.

#### Romberg test

o feet close together;
p arms stretched forwards, palms up;
q eyes open for 30 s; and
r eyes closed for 30 s.

#### Unterberger test

o arms stretched forwards, palms upwards;
p closed eyes; and
q 60 s (50 steps) walking on the spot.

### Questionnaires

Prior to the study, subjects were asked to complete questionnaires regarding their experiences with e-scooters and alcohol. The questions regarding e-scooter driving experience, driving experience under the influence of alcohol and alcohol consumption habits can be seen in the supplementary material (1.).

### Toxicological analyses

BACs were determined according to the current German forensic guidelines [24].

On site, the urine samples were screened for amphetamine, cannabinoids, cocaine, methamphetamine, morphine (opiates), benzodiazepines, buprenorphine, and pregabalin (Drug-Screen Multi 8AQ Test 25 Multi-Dip Tests, nal von minden GmbH, Moers, Germany).

Afterwards, serum from the study participants was analysed as follows: 0.6 ml serum sample was mixed with 20 µl internal standard solution (20 ng/µl camazepam in methanol) and 100 µl sodium carbonate buffer (pH 8.6). The mixture was extracted with 1.2 ml of dichloromethane/diethyl ether (70:30, v/v) and the extract evaporated to dryness under a stream of nitrogen at 60 °C (home-made evaporator). The residue was dissolved in 60 µl acetonitrile/5 mM ammonium formate buffer (13:87, v/v, containing 0.1% formic acid) and analysed by HPLC–PDA-MS/MS (Acquity UPLC-PDA coupled to an Acquity TQ-detector in MRM mode, Waters Europe). An Acquity HSS C18 column (1.8 µm, 2.1 × 150 mm, Waters Europe) was used for HPLC (flow rate 0.45 ml/min). With this LC–MS/MS-MRM method, approximately 230 different drugs and pharmaceuticals can be detected in serum in one run. In addition, substances absorbing ultraviolet light are detected with the PDA detector.

### Evaluation

Demerits were allocated for distinctive features. Regarding driving features, coordinative and cognitive driving features were differentiated. Medical test features were evaluated separately. Features that were considered more relevant for road traffic safety received more demerits. Allocated demerits for specific errors can be seen in the supplementary material (2.).

### Statistical analyses

An “absolute score” (absolute driving performance) was first calculated by adding allocated demerits. Therefore, all unchanged obstacles A, B, C, D, E, F, H were included. Furthermore, for the collective analysis of the four test days, only the first passage of each obstacle during each run was included, as some obstacles had to be passed twice on days 3 and 4. All obstacles were also evaluated separately, leading to an “error score” for each obstacle.

An “individual score” (individual driving performance) was also determined, which resulted from the comparison of the absolute score of the first run (sober) with the absolute score of the respective run while under the influence of alcohol. For this purpose, only obstacles A, B, C, D, E, F, H on their first passage were included.

Subjects with an absolute score of zero points received one point to enable the calculations of all individual scores.

*Individual Score* = *sober absolute score*/*current absolute score*

*Individual Score* = (*sober absolute score* + 1)/(*current absolute score* + 1)

To enhance the baseline data, the collective sober data were always included in the statistical calculations, except when considering the “Influence of alcohol experience on driving performance”.

The result of a statistical test was considered significant if the *p* value was less than 0.05.

## Results

### Subjects

Fifty-seven subjects who consumed alcohol (28 females, 29 males) and 6 sober control subjects (3 females, 3 males) were included in the study.

The median age was 29 years in the alcohol-consuming group (range: 18 to 49 years). The median age for females was 26 years (range: 19 to 48 years) and that for males was 31 years (range: 18 to 49 years).

The control group had a median age of 26 years (women, 27 years, range: 22 to 30 years; men, 26 years, range: 23 to 31 years).

Two female subjects dropped out after the second ride on the fourth day (owing to the hospitalisation of one subject due to an alcohol-related accident off the course and at the request of an escort of the second subject).

### Drug screening

The toxicological analyses of the serum samples showed a positive result for doxylamine (an antihistamine with sedative effects used to treat cold or allergy symptoms) in one test subject and a positive result for fluoxetine (a stimulating antidepressant of the selective serotonin reuptake inhibitor class) in one other person. There was no other intake of drugs or medication with effect of the central nervous system. We see no reason why a previous use of doxylamine or regular use of fluoxetine had a relevant impact on the results of the study.

### Achieved BACs

The median of the achieved maximum BACs was 1.16 g/kg (range of the achieved maximum BACs excluding drop outs: 0.54–1.70 g/kg). Fifteen test subjects did not reach 1.00 g/kg.

### Sex

No difference between females and males in terms of worsening driving performance was found, neither by comparing the results of the “absolute scores” (*p* value: 0.65) nor by comparing the results of the “individual scores” (*p* value: 0.92).

### Influence of driving and alcohol experiences on driving performance

Previous e-scooter driving experience had no influence on the “absolute score” (*p* value: 0.11) or the “individual score” (*p* values: 0.23). In subjects with at least two e-scooter experience points, individual driving performance deteriorated but remained roughly constant with increasing experience points (except an e-scooter driving experience score of 6).

Sixteen percent (10 of 62) of the subjects had previously used an e-scooter under the influence of alcohol.

Neither the “absolute score” (*p* value: 0.13) nor the “individual score” (*p* value: 0.17) showed improved or worsened results in correlation with a drunk driving experience with an e-scooter.

### Romberg test and Unterberger test

#### Insecurity scores

The medical examinations focused on balance. Differences in performance (insecurity scores) on the Romberg test and the Unterberger test, both alone and in combination, were noticeable with increasing BACs. Noticeable differences in Unterberger stepping test performance included increased swaying and stepping insecurity; noticeable differences in Romberg test performance included increased swaying while standing securely with arms outstretched. These differences were reflected in increased demerits in the individual insecurity score. The *p* values for the Romberg test (<0.01) and Unterberger test (<0.01) showed a correlation between an increasing insecurity score and increasing BAC.

The *p* values for the correlation of Romberg performance with the absolute score (<0.01) and individual score (<0.01) showed a correlation between an increasing insecurity score and decreasing driving performance.

The *p* values for the correlation of Unterberger test performance with the absolute score (<0.01) and individual score (<0.01) also showed a correlation between an increasing insecurity score and decreasing driving performance.

#### Turning > 45°

In addition to the insecurity score, differences in turning around from a starting point (Unterberger test) in degrees were noted. A deviation of >45° indicated a positive and conspicuous result. Just a trend for an association between higher BACs and a positive test result was seen (*p* value: 0.15).

### Cognitive errors

#### Remembering a word

**Fig. 4:**
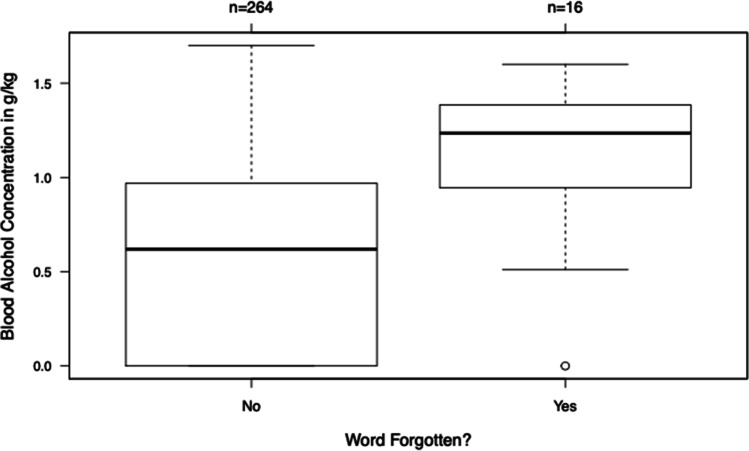
Remembered and forgotten words after each run in comparison to the BAC (y-axis) (*p* value: < 0.01). Boxes contain 50% of the observations. Black lines indicate the respective median. Circles indicate outliers. Satellites indicate the most extreme observations in the range of 1.5 × (interquartile range) to the boxes

In total, the word that was named before each run was forgotten 16 out of 280 times (Figure 4). A correlation between forgetting the word that was named and increasing BAC could be shown (*p* value: <0.01), and the word was mostly forgotten at higher BACs. In addition, a significantly worse driving performance was present if the word could not be remembered (*p* values: absolute score < 0.01; individual score < 0.01).

#### Reaction to light signal during lane driving on days 3 and 4

It could not be shown that the reaction ability was impaired with decreasing driving performance. Only a few subjects reacted incorrectly to the light signal (*p* values: absolute score 0.94; individual score 0.42). Furthermore, no differences in the BAC ranges could be shown (*p* value: 0.20).

### Evaluation of isolated obstacles at increasing BACs

Independent of the BAC, the obstacles gravel bed, three turns with timely directional indication, and thresholds were regularly passed with a low number of demerits or no demerits at all. In conclusion, the *p* values were not exploitable.

The obstacles alley drive and speed track could not be compared to other obstacles, as the number of observations (resp. drives) was too low.

Performance on the narrowing track obstacle showed mainly nonsignificant alcohol-related changes (partially few observations only) in the error score below 1 g/kg. Starting from 1.01 g/kg (e.g. 1.01–1.20 g/kg; *p* value < 0.01), the *p* values were constantly significant (Table 1).

During the gate passage and the slalom obstacles, statistically significant distinctive features in the error score constantly appeared at BACs above 0.80 g/kg (e.g. 0.81–1.00 g/kg: *p* values gate passage < 0.01; slalom < 0.01). With increasing BACs, the *p* values remained constantly significant (Table 1).

**Fig. 5:**
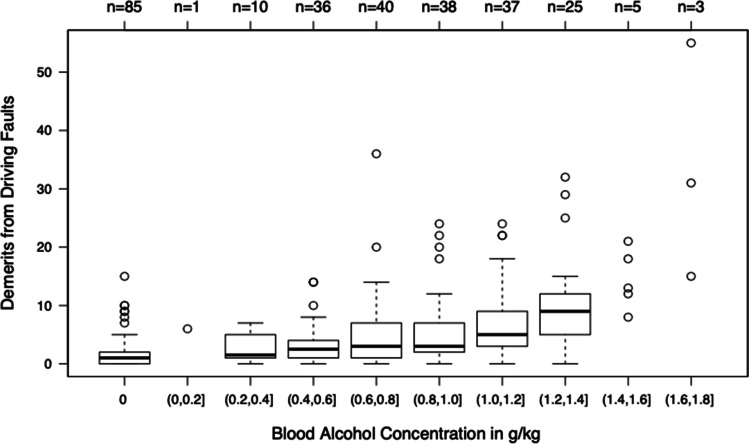
Demerits of the error score for driving in circles counterclockwise at increasing BAC ranges. The figure includes all drives of sober subjects and subjects drinking alcohol. Boxes contain 50% of the observations. Black lines indicate the respective median. Circles indicate outliers. Satellites indicate the most extreme observations in the range of 1.5 × (interquartile range) to the boxes

Driving in circles counterclockwise showed constantly significant results in all BAC ranges above 0.40 g/kg (e.g. 0.41–0.60 g/kg: *p* value < 0.01) (Table 1). The test subjects’ error scores are presented in Figure 5.

### Influence of increasing BACs on the time to pass the obstacles

Whether an increased time to pass the obstacles (regardless of potentially committed errors) correlates with increasing BACs was investigated. The obstacles gate passage, driving in circles counterclockwise, the alley at days 3 and 4, and the speed track at days 3 and 4 required more time to pass with increasing BACs (Table 2, supplementary material).

Furthermore, an increased time to pass the obstacles correlated with an increasing error score. The time needed to pass the narrowing track, gate passage, driving in circles counterclockwise, the alley at days 3 and 4, and the speed track at days 1 and 2 showed highly significant *p* values when compared to the error score (Table 2).

Again, *p* values for the obstacles gravel bed, turns with timely directional indication, thresholds, and alley at days 1 and 2 are only somewhat meaningful, since the achieved error scores of the test subjects were almost always zero (Table 2, supplementary material).

### Individual driving performance at different BAC ranges

In comparison to the driving performance while sober (including the performance of sober control subjects), the driving performance at BACs ranging from 0.21 to 0.40 g/kg exhibited a decrease in the average individual driving performance of approximately 40%. A slower but continuous decrease in the “individual driving performance” at increasing BACs was evident. In the BAC range of 0.81–1.00 g/kg, a mean decrease of 62% was noted. At BACs from 1.01 to 1.20 g/kg, an average decrease of 72% was seen (Table 3; supplementary material).

Absolute and individual scores were significantly altered at low BAC ranges (starting at approximately 0.30 g/kg each; Table 4; supplementary material).

### Habituation and learning in experiment

In the sober collective data, only two subjects showed an improvement in their driving performance on the second drive. The other subjects fluctuated between better and worse driving performance across drives. Compendiously, neither an improvement (e.g. due to habituation to the course) nor a worsening (e.g. due to tiredness) during the trial can be assumed.

## Discussion

To the best of the authors’ knowledge, these experiments provide first-ever e-scooter driving data from prospective real-driving tests.

First, a significant decrease in the individual driving performance already at low BAC ranges was seen. In the BAC range, which is closest to the German threshold of absolute impairment to drive a motor vehicle of 1.10 g/kg (here: 1.01–1.20 g/kg), the individual driving performance decreased strongly.

This severe decrease is highly relevant for road traffic safety because there are indications that e-scooters are used as an alternative to cars for transportation home after alcohol consumption [3]. A decreased “individual score” of this amount can lead to a higher injury risk for both drivers and others on the road.

Second, the isolated obstacles, especially the narrowing track, gate passage, circles counterclockwise, and slalom obstacles, were significantly correlated with high demerits at increasing BACs.

Both the narrowing track and the circles counterclockwise obstacles represent possible situations in which track holding is crucial, e.g. driving on narrow bicycle lanes or turnaround manoeuvres in narrow streets. Significant increases in the “error score” were found in both cases: for the narrowing track obstacle, constantly in the BAC range of 1.01–1.20 (*p* value < 0.01) and for the circles counterclockwise obstacle, constantly in the BAC range as low as 0.41–0.60 (*p* value < 0.01).

The gate passage and slalom obstacles might mimic avoiding pedestrians or their dogs on commonly used ways or parking cars. Here, differences in negotiating the gate passage and slalom obstacles were evident from a minimum BAC of 0.81 g/kg (*p* values gate passage < 0.01; slalom < 0.01). Therefore, drivers of e-scooters with BACs above this range may represent a higher danger for pedestrians.

Third, the e-scooter driving performance under the influence of alcohol was also well reflected in the examination results of the Romberg test and Unterberger test. In a study from Penner and Coldwell in 1958 [25], several medical examinations were discussed and reviewed to evaluate which examination method is appropriate to reflect driving performance under the influence of alcohol. Interestingly, their results are contradictory to ours. However, Penner and Coldwell analysed car driving performance. The equilibrium sense is challenged in car driving much less than in e-scooter driving. This aspect seems crucial. Insofar, one might want to consider equilibrium examinations in the field sobriety report (e.g. so-called Torkelbogen) of e-scooter drivers. We did not carry out all field sobriety tests as they had been assessed by the research group before on a large sample [4]. However, it must be kept in mind that the presented criteria require subjective judgements, and in the present study, the investigators were aware of the amount of alcohol consumed, so the data were gathered in a nonblinded manner.

Fourth, only a few errors were generally seen on other obstacles (gravel bed, turns with timely directional indication, thresholds, one alley), so meaningful results were not obtained for these obstacles.

Unexpected was the low error rate associated with the gravel bed obstacle. We assumed possible falls at higher BACs or at least regular driving difficulties. However, this was not the case. Test subjects drove through the gravel bed rather briskly, which led to fewer uncertainties in the gravel. It is well known that gravel often causes problems when braking and/or turning; therefore, a linear drive-through might not have represented the full threat of a gravel bed.

The thresholds were flattened and not directly shaped like an angular sidewalk. Possibly, this enabled a smoother passage than real lowered curb stones.

The difficulty in the turns with timely directional indication is to signal with one hand while steering with the other hand [17]. Here, only the general execution of an appropriate signal was important, so test subjects could perform the signal in the way that they felt most secure or comfortable. Raising the leg seemed to be much easier than signalling with the hand, which would explain the low error rates.

In general, it should be noted that the subjects were used to driving e-scooters, habituated to the course in the state of soberness, and were fully equipped with a protective gear. Test subjects were aware of the obstacles. Test subjects also knew that there would be a signal they would have to react to right before the alley drive obstacle (days 3 and 4).

With the control group, a learning or exhausting effect could be refuted in the experiment.

Seidl et al. [26] noted that people under the influence of alcohol might classify themselves as fit to drive at a blood alcohol level of even 2.10 g/kg [26]. Our voluntary survey revealed that 16% (10 of 62) had used an e-scooter before under the influence of alcohol. In addition, we were able to show that previous drunk driving experience had no influence on driving performance.

## Conclusions

Even low BACs were demonstrated to pose risks when driving an e-scooter in road traffic. At BACs ranging from 0.21 to 0.60 g/kg, a higher risk of driving dangerously was seen. Referring to the mentioned results from the slalom and gate passage, an increased danger for pedestrians can be assumed at a BAC of at least 0.81 g/kg.

**Table 1:** p values of the sober performance versus the stated BAC range yielded by the error score for the named obstacles. The ranges 0 vs. [0.01–0.20], 0 vs. [1.41–1.60], and 0 vs. [1.61–1.80] are not illustrated, because of low observation numbers in the respective BAC groups (1 to 5 observations only)

BAC	Number of subjects	Narrowing track	Gate passage	Slalom	Circles counterclockwise
0 vs. [0.21–0.40]	10	0.50	0.81	0.02	0.06
0 vs. [0.41–0.60]	36	0.01	0.69	0.18	< 0.01
0 vs. [0.61–0.80]	40	0.12	0.37	0.61	< 0.01
0 vs. [0.81–1.00]	38	0.19	< 0.01	< 0.01	< 0.01
0 vs. [1.01–1.20]	37	< 0.01	< 0.01	< 0.01	< 0.01
0 vs. [1.21–1.40]	25	< 0.01	< 0.01	< 0.01	< 0.01

## Supplementary Information

Below is the link to the electronic supplementary material.

Supplementary file1 (DOCX 25.2 KB)

Supplementary file2 (DOCX 27.7 KB)
