# Supplementary material for: E-scooter driving under the acute influence of alcohol—a real-driving fitness study
Source: Int J Legal Med. 2022 Feb 26;136(5):1281–90. doi: 10.1007/s00414-022-02792-3 (PMC9375743; doi:10.1007/s00414-022-02792-3)
Supplement: Supplementary file 1 — Supplementary file1 (DOCX 25.2 KB) [file 414_2022_2792_MOESM1_ESM.docx]

**Tables and supplementary table material**

| **BAC** | **Number of subjects** | **Narrowing track** | **Gate passage** | **Slalom** | **Circles counterclockwise** |
| --- | --- | --- | --- | --- | --- |
| 0 vs. [0.21-0.40] | 10 | 0.50 | 0.81 | 0.02 | 0.06 |
| 0 vs. [0.41-0.60] | 36 | 0.01 | 0.69 | 0.18 | < 0.01 |
| 0 vs. [0.61-0.80] | 40 | 0.12 | 0.37 | 0.61 | < 0.01 |
| 0 vs. [0.81-1.00] | 38 | 0.19 | < 0.01 | < 0.01 | < 0.01 |
| 0 vs. [1.01-1.20] | 37 | < 0.01 | < 0.01 | < 0.01 | < 0.01 |
| 0 vs. [1.21-1.40] | 25 | < 0.01 | < 0.01 | < 0.01 | < 0.01 |

Table 1: P values of the sober performance versus the stated BAC range yielded by the error score for the named obstacles. The ranges 0 vs. [0.01-0.20], 0 vs. [1.41-1.60] and 0 vs. [1.61-1.80] are not illustrated, because of low observation numbers in the respective BAC groups (1 to 5 observations only).

| **Obstacle** | **p value BAC** | **p value error score** |
| --- | --- | --- |
| *Narrowing track* | 0.91 | 0.01 |
| *Gate passage* | < 0.01 | < 0.01 |
| *Gravel bed** | 0.18 | 0.01 |
| *Circles counterclockwise* | < 0.01 | < 0.01 |
| *Turns with timely directional indication ** | 0.35 | 0.40 |
| *Thresholds** | 0.61 | < 0.01 |
| *Slalom* | 0.99 | 0.82 |
| *Alley * (Days 1 and 2)* | 0.69 | 0.12 |
| *Alley (Days 3 and 4)* | < 0.01 | < 0.01 |
| *Speed track (Days 1 and 2)* | 0.24 | < 0.01 |
| *Speed track (Days 3 and 4)* | 0.02 | 0.09 |

Table 2, supplementary material: P values for time to pass the obstacle in comparison to the increasing BAC and error score. * = low amount of errors. A significant value of the BAC means that the test subjects needed more time to pass the obstacle with increasing BAC. A significant p value for the error score means that the test subject needed more time to pass the obstacle with an increasing error score.

| **BAC** | **Number of subjects** | **Estimate** | **Lower** | **Upper** |
| --- | --- | --- | --- | --- |
| 0🡪 [0.21-0.40] | 10 | 0.59 | 0.38 | 0.91 |
| 0🡪 [0.41-0.60] | 36 | 0.56 | 0.43 | 0.73 |
| 0🡪 [0.61-0.80] | 40 | 0.56 | 0.43 | 0.71 |
| 0🡪 [0.81-1.00] | 38 | 0.38 | 0.29 | 0.49 |
| 0🡪 [1.01-1.20] | 37 | 0.28 | 0.22 | 0.36 |
| 0🡪 [1.21-1.40] | 25 | 0.20 | 0.15 | 0.27 |

Table 3, supplementary material: Individual driving performance under the influence of alcohol. Lower and upper columns represent the bounds of the 95% confidence interval. Baseline: 1.0. The column “Estimate” describes the estimated individual driving performance in comparison to the sober driving performance (“Estimate” = 1), denoted as a decimal value. The ranges 0 vs. [0.01-0.20], 0 vs. [1.41-1.60] and 0 vs. [1.61-1.80] are not illustrated, because of low observation numbers in the respective BAC groups (1 to 5 observations only).

| **BAC** | **Number of subjects** | **Absolute Score** | **Individual Score** |
| --- | --- | --- | --- |
| 0 vs. [0.21-0.40] | 10 | 0.04 | 0.02 |
| 0 vs. [0.41-0.60] | 36 | < 0.01 | < 0.01 |
| 0 vs. [0.61-0.80] | 40 | < 0.01 | < 0.01 |
| 0 vs. [0.81-1.00] | 38 | < 0.01 | < 0.01 |
| 0 vs. [1.01-1.20] | 37 | < 0.01 | < 0.01 |
| 0 vs. [1.21-1.40] | 25 | < 0.01 | < 0.01 |

Table 4, supplementary material: P value of the sober performance versus the stated BAC range yielded by the absolute scores and individual scores. In the range of 0.21 to 0.40 g/kg, a significantly worse driving performance than the sober driving performance was evident. The ranges 0 vs. [0.01-0.20], 0 vs. [1.41-1.60] and 0 vs. [1.61-1.80] are not illustrated, because of low observation numbers in the respective BAC groups (1 to 5 observations only).
