# Supplementary material for: E-scooter driving under the acute influence of alcohol—a real-driving fitness study
Source: Int J Legal Med. 2022 Feb 26;136(5):1281–90. doi: 10.1007/s00414-022-02792-3 (PMC9375743; doi:10.1007/s00414-022-02792-3)
Supplement: Supplementary file 2 — Supplementary file2 (DOCX 27.7 KB) [file 414_2022_2792_MOESM2_ESM.docx]

**Other supplementary material**

1. **E-scooter driving experience**

Numbers in brackets were added and indicated the allocated driving experience score:

- How many times you have ridden an e-scooter before?
- 2 times (1)
- 3 to 5 times (2)
- 6 to 10 times (2)
- More than 10 times (3)
- On average, how often did you ride an e-scooter in the last 6 months?
- Never (0)
- Approximately 1 time per month (1)
- 2-4 times per month (2)
- 2-3 times per week (2)
- 4 or more times per week (3)
- Approximately how long are the average trips you make using an e-scooter?
- < 10 min (0)
- 10-20 min (1)
- >20 min (2)

Total score: Minimum 1 point; Maximum 8 points.

**Driving experience with alcohol**

- Have you ever driven an e-scooter while under the influence of alcohol?
- Yes
- No

**Habitual alcohol consumption**

Subjects were also asked to complete an Alcohol Use Disorders Identification Test (AUDIT) [27, 28]. This test contains ten questions dealing with alcohol use, alcohol dependence, and alcohol abuse. An AUDIT score of 8 points or more indicates a suspected alcohol-related disorder.

1. **The following demerits (in brackets) were allocated:**

**a) Demerits for coordinative errors**

- Narrowing track
  - line touching (1)
  - line crossing (2)
  - line crossing without reentering (5)
- Gate passage
  - pole touching (1)
  - touching pole holder (1)
  - knocking over a pole (3)
  - skipping gate (3)
- Gravel bed
  - entering diagonally (1) per wheel
  - pushing e-scooter into the gravel bed (1)
  - pushing e-scooter while inside the gravel bed (1)
  - sliding out of gravel bed (1)
- Driving in circles counterclockwise
  - touching line (front wheel) (1)
  - crossing line (front wheel) (2)
  - overrunning line (front and rear wheel) (3)
  - each exceeding or forgotten lane (1)
  - demerits for times out of lane
    - measured from the point of time when the front wheel the left lane until the front wheel reentered the lane.
    - total time for obstacle/number of laps
    - percentage of time out of lane calculated per lap
    - 100% = 3 points, e.g., 10% = 0.3 points
    - all points rounded off at the end
- Turning with timely directional indication
  - no sign (2)
  - short-cutting/avoiding the track (2)
- Thresholds
  - incomplete passing (1) per wheel
  - skipping (3) per threshold
- Alley (Days 1 and 2)
  - touching barrel (1)
  - touching pole holder (1)
  - barrel collision (4)
  - skipping alley (5)
- Alley (Days 3 and 4)
  - touching barrel (1)
  - barrel collision (4)
  - skipping alley (5)
- Slalom ride with decreasing spacing
  - touching cone (1) per wheel
  - running over cone (2) per wheel
  - skipping cone (2) per wheel
  - skipping whole slalom ride with decreasing spacing (5)
- Speed track (Days 1 and 2)
  - touching stop line (1)
  - stopping too early (1)
  - overrunning the stop line (2)
- Speed track (Days 3 and 4)
  - touching line (1)
  - foot not put on the ground (1)
  - incomplete stopping (1)
  - stopping too early (1)
  - overrunning stop line while breaking with one wheel (2)
  - overrunning stop line with both wheels (3)
- Additional could be added for each obstacle, if
- test subjects stabilized their drive with one foot on the ground (1)
- descended from the e-scooter (2)

**b) Demerits for cognitive errors**

- Alley (Days 3 and 4)
  - - - reaction to light signal not correct (3)
- Was the word remembered correctly?
  - - - yes
      - no (no demerits allocated, separate evaluation).

**c) Demerits from medical tests**

**Romberg Test**

- - safe, 0
  - slight uncertainty (1)
  - strong swaying (2)
  - termination by the test subject (3)
  - termination by the examiner (3)

**Unterberger Test**

- - deviation in degrees >45°; <45°
  - safe (0)
  - slight uncertainty (1)
  - strong swaying (2)
  - termination by the test subject (3)
  - termination by the examiner (3)

Allocated points for the determination of the driving experience score and AUDIT score are mentioned separately.
